# Immunity to Tick-Borne Encephalitis Virus NS3 Protein Induced with a Recombinant Modified Vaccinia Virus Ankara Fails to Afford Mice Protection against TBEV Infection

**DOI:** 10.3390/vaccines12010105

**Published:** 2024-01-20

**Authors:** Mareike Kubinski, Jana Beicht, Thomas Gerlach, Amare Aregay, Albert D. M. E. Osterhaus, Alina Tscherne, Gerd Sutter, Chittappen Kandiyil Prajeeth, Guus F. Rimmelzwaan

**Affiliations:** 1 Research Center for Emerging Infections and Zoonoses, University of Veterinary Medicine Hannover, Foundation, Bünteweg 17, 30559 Hannover, Germany; mareike.kubinski@gmail.com (M.K.); jana.beicht@tiho-hannover.de (J.B.); thomasgerlach86@outlook.de (T.G.); amare.aregay@tiho-hannover.de (A.A.); albert.osterhaus@tiho-hannover.de (A.D.M.E.O.); prajeeth.chittappen.kandiyil@tiho-hannover.de (C.K.P.); 2 Division of Virology, Institute for Infectious Diseases and Zoonoses, Ludwig Maximilian University Munich, Sonnenstraße 24, 85764 Oberschleißheim, Germany; a.tscherne@lmu.de (A.T.);; 3 German Center for Infection Research (DZIF), Partner Site Munich, 80802 Munich, Germany

**Keywords:** TBEV, MVA, NS3, vaccination, antibodies, T cells

## Abstract

Tick-borne encephalitis (TBE) is a serious neurological disease caused by TBE virus (TBEV). Because antiviral treatment options are not available, vaccination is the key prophylactic measure against TBEV infections. Despite the availability of effective vaccines, cases of vaccination breakthrough infections have been reported. The multienzymatic non-structural protein 3 (NS3) of orthoflaviviruses plays an important role in polyprotein processing and virus replication. In the present study, we evaluated NS3 of TBEV as a potential vaccine target for the induction of protective immunity. To this end, a recombinant modified vaccinia virus Ankara that drives the expression of the TBEV NS3 gene (MVA-NS3) was constructed. MVA-NS3 was used to immunize C57BL/6 mice. It induced NS3-specific immune responses, in particular T cell responses, especially against the helicase domain of NS3. However, MVA-NS3-immunized mice were not protected from subsequent challenge infection with a lethal dose of the TBEV strain Neudoerfl, indicating that in contrast to immunity to prME and NS1, NS3-specific immunity is not an independent correlate of protection against TBEV in this mouse model.

## 1. Introduction

The genus *Orthoflavivirus* within the family *Flaviviridae* comprises important human pathogens like dengue virus (DENV), Zika virus (ZIKV) and tick-borne encephalitis virus (TBEV) [1,2]. TBEV is the causative agent of tick-borne encephalitis (TBE), the most important tick-transmitted disease in Europe and parts of Asia, with up to 15,000 clinical cases reported worldwide each year [3]. The clinical picture of TBE patients varies and severe cases can suffer from neurological symptoms affecting the central and autonomic nervous systems [4,5]. There is no treatment for TBE other than symptomatic, and antiviral drugs have not yet been approved in Europe [6]. Although the use of inactivated TBE vaccines has reduced the number of clinical TBE cases [7,8], vaccination breakthrough infections are reported regularly [9,10,11,12]. In addition, the number of human TBE cases is increasing in Europe [13] and novel foci of TBEV cases have been reported in recent years [14,15,16,17].

The genomic RNA (single-stranded, positive-sense) of TBEV encodes one polyprotein, which is cleaved co- and post-translationally into single proteins by proteases of the host and TBEV itself (structural proteins: capsid (C), pre-membrane (prM), envelope (E); non-structural (NS) proteins: NS1, NS2A, NS2B, NS3, NS4A, NS4B, NS5). Whereas structural proteins are components of the virion, NS proteins play a role in orthoflavivirus replication and immune evasion [18]. Among NS proteins, NS3 is of special interest due to its central role in the orthoflavivirus replication cycle. The NS3 protein plays a role in polyprotein processing and virus replication due to its enzymatic activities and has further immunomodulatory properties. The N terminus of NS3 comprises the serine protease which, together with NS2B as co-factor, cleaves specific sites within the orthoflaviviral polyprotein [19,20]. One mutation within NS3 in the vicinity of the catalytic region of the protease is thought to alter the neuroinvasiveness of a TBEV strain [21]. In the C terminal region, there is the helicase/NTPase, which is involved in unwinding dsRNA and is important for orthoflavivirus replication [19,20]. The C terminus of TBEV NS3 also contains a 5′-RNA triphosphatase [22]. Furthermore, orthoflaviviral NS3 is one of the major targets for the induction of virus-specific T cell responses [23,24]. In hospitalized TBE patients, CD8^+^ T cells directed to NS3 have been detected early after clinical onset and have been of the effector and memory phenotypes [25,26].

We hypothesized that the use of NS3 as candidate vaccine antigen could result in the induction of specific T cell responses which could afford a certain degree of protection against challenge infection with TBEV. Previous studies performed with NS3-based orthoflavivirus candidate vaccines have not been conclusive so far. Vaccination of mice with plasmid DNA encoding TBEV NS3 failed to protect against TBEV challenge infection [27]. In contrast, DNA-based vaccination with full-length DENV NS3 or its helicase domain protected mice against lethal DENV challenge infection but not against the development of clinical signs [28]. Analysis of NS3 vaccine-induced immune responses indicated that NS3-specific cell-mediated immunity is the main correlate of partial protection against DENV infection [28,29].

Therefore, in the present study, we investigated immunity to TBEV NS3 with emphasis on the induction of NS3-specific T cell responses and assessed its protective efficacy. To this end, we cloned the TBEV NS3 gene into the replication-deficient poxviral vector modified vaccinia virus Ankara (MVA) to generate a recombinant MVA that drives the expression of the NS3 gene (MVA-NS3). MVA has been shown to be safe and able to induce strong antibody and T cell responses to the expressed protein of interest [30]. MVA-based candidate vaccines for several viruses, like influenza virus, coronaviruses and human immunodeficiency virus have already been tested in clinical trials [31,32,33,34]. Recently, we constructed and tested recombinant MVA candidate vaccines expressing the prM-E and NS1 TBEV proteins, respectively. With these recombinant MVAs, we were able to induce E- and NS1-specific antibody and T cell responses which afforded complete or partial protection, respectively, against lethal challenge infection in mice [35,36].

Following the in vitro characterization of MVA-NS3, its immunogenicity and protective efficacy was investigated in C57BL/6 mice. To this end, mice were prime-boost vaccinated with MVA-NS3, and NS3-specific antibody and T cell responses were determined. However, a vaccination challenge experiment showed that immunization with MVA-NS3 did not afford mice protection against infection with a lethal dose of the TBEV strain Neudoerfl (European subtype). Based on these data, it was concluded that NS3-specific antibodies and T cells are not independent correlates of protection and inclusion of NS3 in future improved TBE vaccines does not seem warranted.

## 2. Materials and Methods

### 2.1. Cells and Viruses

Primary CEF cells (prepared from 10–11-day-old chicken embryos (specific pathogen-free eggs from VALO BioMedia GmbH, Osterholz-Scharmbeck, Germany)), HeLa cells and A549 cells were cultured as described previously [35]. Wildtype MVA (MVA F6 isolate) and recombinant MVA-GFP (containing green fluorescent protein (GFP) gene in deletion site III under transcriptional control of the late promotor P11 of vaccinia virus (VACV)) [37] were used. European TBEV subtype strain Neudoerfl was obtained from Dr. G. Dobler, the Department of Microbiology of the German Armed Forces, Munich, Germany. Cells and viruses tested negative for mycoplasma.

### 2.2. Generation of MVA-NS3 and In Vitro Characterization

**Figure 1:**
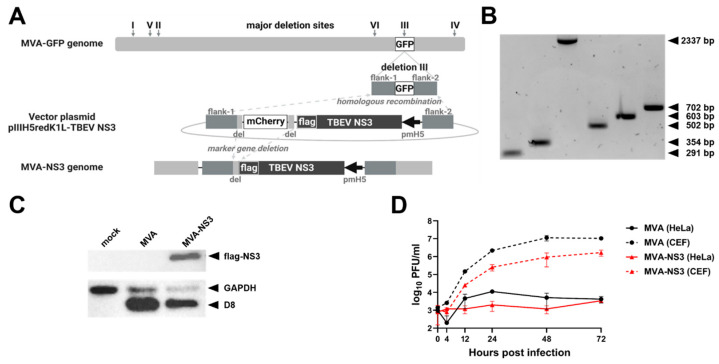
In vitro characterization of MVA-NS3. (**A**) Generation of recombinant MVA-NS3 by homologous and intragenomic homologous recombination. The TBEV NS3 gene is expressed under transcriptional control of VACV early/late promoter pmH5. (**B**) Fingerprint PCR using primer sets specific to the six major deletion sites of MVA (I: 291 bp, II: 354 bp, III: 447 bp, IV: 502 bp, V: 603 bp, VI: 702 bp). Insertion of the NS3 gene into deletion site III was confirmed (III: 2337 bp). (**C**) Western blot analysis of lysates prepared with HeLa cells 24 h after infection with MVA or MVA-NS3 (MOI 5) using antibodies against the flag tag, VACV D8 and GAPDH. Mock-infected cells were included as negative control. (**D**) Replication kinetics of MVA (black) and MVA-NS3 (red) in primary CEF cells (dotted lines) and HeLa cells (solid lines) (MOI 0.05).

The nucleotide sequence of TBEV Neudoerfl NS3 (GenBank: U27495.1) was modified in silico by adding the Kozak sequence followed by a flag tag prior to the NS3 sequence. The synthesized sequence (GenScript Biotech Corp, Piscataway, NJ, USA) was cloned into the MVA transfer vector plasmid pIIIH5redK1L under transcriptional control of the VACV early/late promoter pmH5 [37] resulting in pIIIH5redK1L-TBEV NS3. Recombinant MVA with integrated NS3 sequence in deletion site III (MVA-NS3) was generated by homologous and intragenomic homologous recombination using the modified standard protocol [37] (Figure 1A). MVA-NS3 was propagated in primary CEF cells and concentrated as described previously [35,36].

To confirm the correct insertion of the NS3 gene into deletion site III, PCRs specific to the six major deletion sites within the MVA genome were performed according to a slightly modified standard protocol [37] and as described previously [35,36]. The identity of the insert was confirmed by Sanger sequencing (Microsynth AG, Balgach, Switzerland).

NS3 expression was demonstrated in HeLa cells using Western blot analysis. To this end, cells were infected with MVA-NS3 or MVA (multiplicity of infection [MOI] 5) or left untreated (mock). Cell lysates were harvested and Western blot was performed as described previously [35,36]. As primary antibodies, polyclonal rabbit D8L antibody (1:2000, Cusabio, Houston, TX, USA), monoclonal mouse ANTI-FLAG^®^ M2 antibody (1:1000, Sigma-Aldrich, St. Louis, MO, USA) and GAPDH (D16H11) XP^®^ Rabbit mAb #5174 (1:3000, Cell Signaling Technology^®^, Danvers, MA, USA) were used. Goat anti-rabbit IgG (H + L) HRP (1:5000, Invitrogen™, Carlsbad, CA, USA) and goat anti-mouse IgG (H + L) HRP (1:5000, Invitrogen™, Carlsbad, CA, USA) were used as secondary antibodies.

Multistep growth kinetics of MVA-NS3 and MVA were performed on primary CEF and HeLa cells as described previously [35,36].

### 2.3. Mouse Studies

#### 2.3.1. Ethical Statement

All mouse experiments were performed at the University of Veterinary Medicine Hannover, Foundation, Hannover, Germany, in strict compliance with the German Animal Welfare Law and EU directive on animal testing 2010/63/EU. The animal protocol was approved by the Lower Saxony State Office for Consumer Protection and Food Safety under the approval number 33.8-42502-04-20/3437.

#### 2.3.2. Mice

Female C57BL/6JOlaHsd (C57BL/6) mice (Envigo RMS GmbH, Venray, The Netherlands) were housed in individually ventilated cages type SEALSAFE PLUS GM500 or IsoCage N Biocontainment System (Tecniplast, Hohenpeißenberg, Germany) with sterilized food and water ad libitum. Prior to the start of experiments, the mice were acclimatized and habituated for at least one week. All treatments were performed under isoflurane anesthesia.

#### 2.3.3. Immunogenicity Study

C57BL/6 mice (6–8 weeks old, *n* = 4 per group) were vaccinated twice with an interval of four weeks (prime-boost) with TBS (negative control; intramuscular (i.m)), 10^7^ plaque-forming units (PFU) of non-recombinant MVA (vector control; i.m.), 10^7^ PFU MVA-NS3 (i.m.) or 0.816 µg FSME-IMMUN^®^ 0.5 mL (Pfizer Pharma GmbH, Berlin, Germany, lot number EM2898; positive control; subcutaneous (s.c.)). Data of mice vaccinated with FSME-IMMUN^®^ (positive control) and MVA (empty vector control) were taken from a shared study which was performed concurrently (same approval number, [35]) with the mice that received MVA-NS3. This way, the number of animals was minimized to adhere to the 3R concept (replacement, reduction and refinement). A clinical score sheet including outer appearance, behavior, movement, body weight and neurological signs was used to assess the clinical scores of the mice on a weekly basis. Twenty-eight days post boost vaccination, blood was collected by retrobulbar punction, mice were euthanized by cervical dislocation and spleens were collected. Spleens were processed and resuspended as described previously [35,36,38].

#### 2.3.4. Protective Efficacy Study

C57BL/6 mice (6–8 weeks old, *n* = 12 per group) were prime-boost vaccinated and scored weekly as described above. Data of MVA-vaccinated mice (empty vector control) were taken from a shared study, which was performed in parallel and simultaneously (same approval number, [35,36]) with the mice that received MVA-NS3. Fifty-six days post prime immunization and before infection with TBEV, blood was collected by puncturing the *Vena facialis*. For inoculation, 5.4 × 10^3^ tissue culture infectious dose 50% (TCID_50_) TBEV Neudoerfl was injected s.c. at day 56 post prime immunization. Mice were monitored daily according to a clinical score sheet. At eight days post infection (dpi), half of the group (*n* = 6) was sacrificed and the other mice (*n* = 6) stayed in the experiment until the pre-defined humane endpoint (HEP) or study endpoint (16 dpi) was reached. When animals were taken out of the experiment, blood was collected as described before prior to euthanasia by cervical dislocation. The brains (left hemisphere), spinal cords (cervical region), spleens, ilea and colons (both size of rice-corn) were collected and homogenized as described previously [35,36,38].

### 2.4. Humoral Immune Response

#### 2.4.1. Virus Neutralization Assay

Determination of virus-neutralizing (VN) antibodies was performed on A549 cells based on the development of cytopathic effects (CPEs) as described previously [35,36]. VN titer (VNT_100_) was determined as the reciprocal of the highest dilution with no CPEs.

#### 2.4.2. Analysis of NS3-Specific Antibodies

A luciferase immunoprecipitation system (LIPS) assay targeting domain III of the TBEV Neudoerfl NS3 protein (NS3-DIII) was performed with sera (30 min/56 °C) as described before [39]. NS3-DIII-luciferase fusion protein was kindly provided by Dr. I. Steffen, University of Veterinary Medicine Hannover, Germany. Values above 2500 relative light units were considered positive. Due to insufficient volume of harvested serum of some mice of the protective efficacy study (for TBS 4 mice, for MVA 3 mice and for FSME-IMMUN^®^ 2 mice were missing), these serum samples were not tested in LIPS assay.

### 2.5. Cellular Immune Response

#### 2.5.1. Restimulation of Spleen Cells

For splenocyte restimulation, 15 mer peptides overlapping by eleven amino acids covering the entire NS3 protein of TBEV Neudoerfl (UniProtKB: P14336) were synthesized and resolved in DMSO (Hybri-Max™, Sigma-Aldrich, St. Louis, MO, USA). Three peptide pools were prepared (NS3_1–215_, NS3_205–419_, NS3_409–621_). Splenocytes were restimulated as described previously [35,36].

#### 2.5.2. IFN-γ ELISpot Assay

Virus-specific T cell responses were determined using a mouse IFN-γ ELISPot assay (IFN-γ ELISpot Plus kit (ALP, Mabtech, Nacka Strand, Sweden) according to manufacturer’s instructions. For analysis, ImmunoSpot^®^ S6 Ultimate Reader and ImmunoSpot^®^ software (version 7.0.20.1, Cellular Technology Ltd., Shaker Heights, OH, USA) were used. An average of triplicates was calculated, background values of medium with 0.5% DMSO were subtracted and data were extrapolated to IFN-γ spot-forming cells (SFC) per 10^6^ splenocytes.

#### 2.5.3. Flow Cytometry

For the last four hours of restimulation, splenocytes were incubated with 10 µg/mL brefeldin A (Sigma-Aldrich, St. Louis, MO, USA). Splenocytes were stained, fixed and permeabilized as described before [35,36]. Antibodies to CD3e, CD4, CD8a, IFN-γ and Granzyme B (BioLegend^®^, San Diego, CA, USA) were purchased from eBioscience™ (Invitrogen™, Carlsbad, CA, USA) if not otherwise stated. Stained cells were resuspended in 1x PBS. Data acquisition was performed using BD LSR Fortessa X-20 with BD FACSDiva (version 9.0, BD Biosciences, Franklin Lakes, NJ, USA). For analysis, FlowJo™ (version 10.8.1, BD Biosciences, Franklin Lakes, NJ, USA) was used. Background values of medium with 0.5% DMSO were subtracted.

### 2.6. Analysis of TBEV RNA Copy Numbers in Organs

RNA isolation of cleared organ homogenates, real-time quantitative reverse transcriptase PCR (RT-PCR) and analysis of data were conducted as described previously [35,36]. The TBEV RNA standard was kindly provided by Dr. S. Becker, University of Veterinary Medicine Hannover, Germany.

### 2.7. Statistics

For pair-wise comparison of T cell responses, data were analyzed using an unpaired *t*-test. For comparison of RNA copy numbers in organs between different groups, one-way ANOVA was used. Survival data were analyzed using a log-rank test. A *p*-value of ≤0.05 was considered significant. Statistics were performed with GraphPad Prism (version 9.0.0).

## 3. Results

### 3.1. MVA-NS3—Generation and In Vitro Characterization

For its expression, the TBEV NS3 gene was inserted into deletion site III of the MVA genome by homologous recombination, resulting in recombinant MVA-NS3 (Figure 1A). The insertion of the entire NS3 nucleotide sequence and absence of mutations were confirmed by fingerprint PCR targeting all six major deletion sites of MVA (Figure 1B) and Sanger sequencing. In addition, unimpaired expression of NS3 in MVA-NS3-infected cells was confirmed by Western blot analysis using whole cell lysates (Figure 1C). The replication deficiency of non-recombinant MVA and MVA-NS3 was confirmed in non-permissive human HeLa cells. In contrast, both viruses replicated to high titers in permissive CEF cells (Figure 1D).

### 3.2. Vaccination with MVA-NS3 Induces TBEV NS3-Specific T Cells

Next, we assessed the immunogenicity of MVA-NS3. To this end, C57BL/6 mice were immunized twice over an interval of four weeks with TBS (mock), non-recombinant MVA (MVA), FSME-IMMUN^®^ or MVA-NS3. Four weeks after the second immunization, sera and splenocytes were tested for the presence of specific antibodies and T cells, respectively. As expected, only mice vaccinated with FSME-IMMUN^®^ developed virus-neutralizing (VN) antibodies (266.6–640 VNT_100_) (Table 1). NS3-DIII-specific antibodies were detectable by LIPS assay in two out of sixteen mice immunized with MVA-NS3 (Table 1).

**Figure 2:**
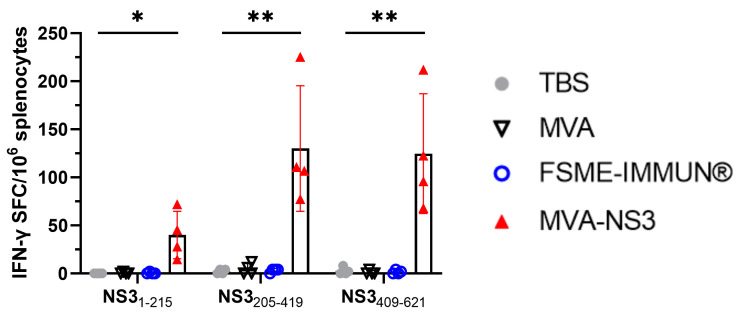
NS3-specific T cell responses. Mice were immunized twice with TBS (gray circle), MVA (open black triangle), FSME-IMMUN^®^ (open blue circle) or MVA-NS3 (red triangle). Four weeks after the second vaccination, splenocytes were isolated and restimulated with TBEV NS3 peptide pools NS3_1–215_, NS3_205–419_ and NS3_409–621_. The graph shows the frequencies of specific T cells determined using IFN-γ ELISpot assay. The frequencies of IFN-γ spot-forming cells (SFC) per 10^6^ splenocytes are shown for individual mice after subtraction of background values. The bars represent group means with standard deviations. Statistically significant differences between TBS- and MVA-NS3-vaccinated mice are indicated (* *p* ≤ 0.05, ** *p* ≤ 0.01).

To determine the NS3-specific T cell response upon immunization, splenocytes were restimulated with three NS3 peptide pools spanning the entire NS3 protein of TBEV or empty MVA vector. As shown by IFN-γ ELISpot assay, vaccination with MVA-NS3 induced T cell responding to NS3 peptide pool restimulation, in particular to NS3_205–419_ and NS3_409–621_ with frequencies of IFN-γ-producing cells of up to 225 SFC per 10^6^ splenocytes (Figure 2). As expected, immunization with MVA and MVA-NS3 also induced strong T cell responses to the MVA vector with frequencies of IFN-γ-producing cells of up to 1200 SFC per 10^6^ splenocytes. In some high-responder mice, further analysis via flow cytometry showed that the majority of NS3-specific IFN-γ-producing cells were CD4^+^ T cells. Furthermore, granzyme B-producing CD4^+^ T cells were observed after stimulation with NS3_1–215_ and NS3_409–621_ peptide pools. In addition, granzyme B^+^ CD8^+^ T cells were also observed against the latter pool.

### 3.3. Vaccination with MVA-NS3 Fails to Protect Mice from TBEV Infection

**Figure 3:**
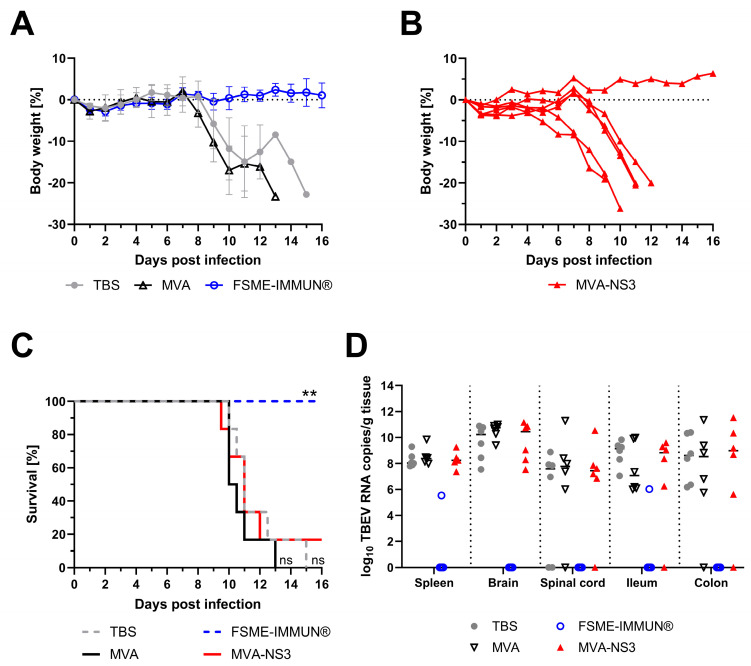
Vaccination with MVA-NS3 does not afford protection from TBEV infection. (**A**) Body weight loss after challenge infection with TBEV Neudoerfl. Results are expressed as the mean of each group ± standard deviation. Groups of six mice received TBS (mock-vaccinated, gray circle), MVA (empty vector, open black triangle) or FSME-IMMUN^®^ (open blue circle). (**B**) Body weight change of individual mice vaccinated with MVA-NS3 after challenge infection with TBEV Neudoerfl. (**C**) Survival of mice after challenge infection with TBEV Neudoerfl (TBS: gray dotted line; MVA: black line; FSME-IMMUN^®^: blue dotted line; MVA-NS3: red line). Kaplan–Meyer curves were analyzed using log-rank test compared to MVA-NS3 (ns = not significant, ** *p* ≤ 0.01). (**D**) TBEV RNA copies were quantified with real-time quantitative RT-PCR in the indicated organs from vaccinated and TBEV-challenged mice sacrificed at 8 dpi (*n* = 6). Lines depict median values. No significant differences were seen. Mice were immunized with TBS (gray circle), MVA (open black triangle), FSME-IMMUN^®^ (open blue circle) or MVA-NS3 (red triangle).

Because vaccination with MVA-NS3 induced NS3-specific T cell responses, we next tested whether immunity induced with two doses of MVA-NS3 could afford protection against infection with a lethal dose of TBEV strain Neudoerfl. Starting at 7–8 dpi, negative control animals that had been mock-vaccinated or that had received the empty MVA vector lost body weight (Figure 3A). These animals displayed severe clinical signs of infection, including reduced spontaneous movement, hunched back posture, dull fur and neurological signs. All negative control animals reached the pre-defined HEP before 16 dpi (Figure 3C). In contrast, all mice vaccinated with FSME-IMMUN^®^ maintained their body weight and did not develop clinical signs until the study endpoint (Figure 3A,C). Vaccination with MVA-NS3 did not protect mice against body weight loss (Figure 3B) and the development of clinical signs which were similar to those in the negative control animals. Consequently, these five mice reached the HEP and the survival rate was not statistically different from the negative control groups (Figure 3C). Upon dissection, macroscopical lesions presenting as segmental distensions were observed in the gastrointestinal tract in all negative control mice that had been mock-vaccinated (TBS) or that had received the empty MVA vector and in five out of six MVA-NS3-vaccinated mice. In contrast, all mice vaccinated with FSME-IMMUN^®^ did not display any lesions in the gastrointestinal tract.

To test whether MVA-NS3-induced immunity could restrict TBEV replication and viral spread to the periphery, central nervous system and intestine, half of the mice of each group were sacrificed at 8 dpi to determine the viral loads in various organs by real-time quantitative RT-PCR (Figure 3D). High TBEV RNA copy numbers were detectable in the spleens, brains, spinal cords, ilea and colons of mock- and empty MVA-vaccinated mice. In contrast, immunization with FSME-IMMUN^®^ completely protected against TBEV replication and viral spread, except for two mice which had low TBEV RNA copy numbers in the spleen or ileum, respectively. Immunization with MVA-NS3 did not prevent TBEV replication in most of the respective organs and high viral loads were detected in the spleens and brains of all six mice as well as in the spinal cords, ilea and colons of five out of six mice in this group.

## 4. Discussion

Previous studies indicated that the inclusion of NS proteins like NS1 or NS3 in vaccine formulations contributes to improved vaccination outcomes [40,41,42,43] and that immunity to NS proteins is even an independent correlate of protection [28,36,44,45]. Therefore, in the present study, we aimed to investigate immunity to TBEV NS3 and its protective efficacy against TBEV infection. For the delivery of TBEV NS3, we used the poxviral vector MVA, which is known for its capacity to induce humoral and cellular immune responses and its excellent safety profile [30]. Indeed, with MVA-NS3, NS3-specific T cells were induced, as demonstrated with IFN-γ ELISpot assay and flow cytometry. We hypothesized that NS3-specific cell-mediated immunity could afford protection by limiting virus replication, as was demonstrated for DENV infections [29]. However, challenge infection of MVA-NS3-immunized mice indicated that NS3-specific T cells were insufficient as an independent correlate of protection.

Prime-boost vaccination with MVA-NS3 induced weak NS3-specific antibody responses, as demonstrated with NS3-DIII LIPS assay. Only two out of sixteen mice had detectable antibody responses. It should be mentioned that only the presence of NS3-DIII-specific antibodies was tested in the LIPS assay, which may have rendered this assay relatively insensitive, and it cannot be excluded that more mice seroconverted and developed antibodies directed to other domains of the protein. However, NS3 proteins of TBEV and other mosquito-borne orthoflaviviruses seem to be poorly immunogenic compared to other orthoflaviviral proteins [27,28,39]. As expected, immunization with MVA-NS3 did not induce VN antibodies, in contrast to vaccination with the licensed vaccine FSME-IMMUN^®^, which is based on formaldehyde-inactivated virus particles and was included in the present study as a positive control. In contrast to the weak antibody responses, immunization with MVA-NS3 induced robust cellular responses. In IFN-γ ELISpot assay, the highest response detected was to TBEV NS3 NS3_205–419_ and NS3_409–621_ peptide pools, spanning mainly the C terminal helicase region of NS3 (based on UniProtKB: P14336). Of interest, also in humans, the helicase is the preferred target of NS3-specific T cell responses after infection with other orthoflaviviruses like ZIKV and DENV [46]. Flow cytometry largely confirmed the data obtained in the IFN-γ ELISpot assay and also indicated that most of the IFN-γ producing cells were CD4^+^ T cells. Some of these also produced granzyme B, indicating that they may also exert lytic activity against virus-infected cells. It has been shown that NS3 of TBEV contains epitopes recognized by human CD8^+^ T cells and that these epitopes are largely conserved among different TBEV subtypes [25,26]. Therefore, NS3 is considered a target for cross-reactive T cells as described for other orthoflaviviruses like ZIKV and DENV [24,46]. However, the NS3-specific CD8^+^ T cell response induced by vaccination with MVA-NS3 in C57BL/6 mice was modest, if detectable at all, in the present study.

Although immunization with MVA-NS3 elicited virus-specific cellular immune responses, immunized mice were not protected from virus replication in the spleen and brain. Apart from one mouse, high viral loads were also detected in the spinal cord and in the gastrointestinal tract of the mice, which developed severe disease and succumbed to infection with TBEV strain Neudoerfl. Pathological changes in the gastrointestinal tract may be one of the critical factors responsible for the overt occurrence of clinical signs and body weight loss, a phenomenon that was observed previously [36,47]. Of interest, our findings are in concordance with another study showing that immunization with plasmid DNA encoding NS3 of TBEV strain Sofjin failed to protect BALB/c mice against homologous TBEV challenge infection [27]. In contrast, vaccines based on NS3 derived from other orthoflaviviruses like DENV and ZIKV at least afforded mice a certain degree of protection against infection with the respective viruses [28,44]. Although NS3-specific immunity afforded a certain degree of protection against some orthoflaviviruses, it was not an independent correlate of protection against TBEV infection, unlike immunity to prME and NS1, as we demonstrated recently [35,36]. Although we cannot exclude that NS3-specific T cells may contribute to protective immunity against TBEV, their role seems redundant in contrast to the presence of humoral and cellular immunity to prME and NS1 [35,36]. 

Collectively, we have demonstrated that following immunization with recombinant MVA expressing the TBEV NS3 gene, NS3-specific T cell responses are induced. However, the induction of NS3-specific immunity is insufficient to significantly reduce virus replication in the respective organs, prevent severe disease and improve survival rates after challenge infection with TBEV strain Neudoerfl. Therefore, NS3 is not considered a promising component of next-generation TBE vaccines, and other viral proteins, like prME and NS1, should be preferred.

## Figures and Tables

**Table 1:** VN and NS3-DIII-specific antibody response. Mouse sera collected four weeks after the second immunization were tested for the presence of VN antibodies by VN assay (4 mice from the immunogenicity study) and NS3-DIII-specific antibodies by LIPS assay (12–16 mice from immunogenicity and protective efficacy studies).

	Positive/Total Mice Tested	
Treatment Group	VN Antibodies	NS3-DIII-Specific Antibodies
TBS	0/4	0/12
MVA	0/4	0/13
FSME-IMMUN^®^	4/4	0/14
MVA-NS3	0/4	2/16

## Data Availability

The data presented in this study are available on request from the corresponding author.
